# The Long-Term Impact of Resilience-Building Interventions on Nurses: A Narrative Review of the Quantitative Evidence and Its Implications for Critical Care Nurses

**DOI:** 10.3390/healthcare13030274

**Published:** 2025-01-30

**Authors:** Maria Kyranou, Maria Karanikola

**Affiliations:** Department of Nursing, School of Health Sciences, Cyprus University of Technology, Limassol 3041, Cyprus; maria.kyranou@cut.ac.cy

**Keywords:** interventions, longitudinal, nurses, resilience, long-term follow-up, well-being, critical care

## Abstract

Background: To minimize systematic bias, long-term follow-up is essential to assess the effect of resilience-building interventions. However, research focuses on the short-term period immediately following these interventions. Objectives: We investigated the long-term impact of resilience-building interventions on nurses, as measured via RCTs. Methods: A narrative review based on a systematic literature search (September–15 November 2024) using the keywords “Resilience/Psychological, Adaptation/Psychological, nurses, randomized controlled trial, follow-up” in the EBSCOhost, MEDLINE, ProQuest, Google Scholar, PubMed, and Scopus databases was applied. Results: A total of 38 studies were identified. Of these, only six encompassed long-term follow-up assessment after resilience-building interventions, also meeting the inclusion criteria for this review. Two of them focused on critical care nurses. Intervention durations ranged from 8 to 12 weeks, with shorter interventions also included (90 min lecture on stress, 3 h sensory awareness class). These data suggest that resilience improvements may become apparent 3 months post intervention, even when no immediate improvement is observed upon program completion, highlighting the importance of timing in the assessment process. Conclusions: These findings provide valuable insights for researchers designing resilience programs in critical care environments. Selecting appropriate assessment tools and conducting measurements at multiple time points may be as crucial as the interventions themselves in determining their effectiveness. This is clinically meaningful since it may inform providers of resilience programs for the time frame during which they need to be applied. Potentially, future research can explore what characteristics these interventions need to have in order to be effective longitudinally.

## 1. Introduction

Improving personal resilience among nurses in hospital settings is essential for the well-being and efficiency of employees and the safety of patients [1]. Nurses often face significant work-related stressors, which may negatively impact their physical and mental health, as well as the quality of care they provide [2]. These challenges are especially pronounced in critical care settings, where nurses encounter traumatic events and morally complex decisions, with implications for their overall well-being [3,4]. Consequently, burnout syndrome and post-traumatic stress disorder, anxiety, and depressive symptoms are commonly reported among them [4,5,6]. These issues highlight the urgent need for targeted interventions to promote personal resilience, particularly for critical care nurses [7].

Several resilient-building initiatives have been developed to support nurses in coping with the emotional and physical demands of caring for critically ill patients [1,8]. However, most of these focus primarily on individual traits, often overlooking external contextual influences, such as the workplace environment, organizational support, and institutional policies, which constitute a crucial role in promoting personal resilience [1]. At the same time, the critical aspect of the duration of the intervention is frequently ignored [1].

These are even more important when one considers recent conceptual frameworks, which suggest that personal resilience is better understood as an adaptative outcome in response to prolong or intense stress, rather than a static personality trait [7]. This dynamic perspective shows the interplay between personal characteristics and external factors in shaping an individual’s capacity to recover from stress, marking a paradigm shift in the conceptualization of resilience [9].

Importantly, adaptation processes require time, making it vital to consider the temporal dimensions of resilience-building programs. Evaluating interventions should also account for factors that facilitate or hinder adaptation. Tailoring educational programs to align with participants’ needs, particularly based on the time elapsed since an intervention, could significantly enhance their effectiveness and relevance.

Despite a growing body of literature on resilience interventions in nursing, no studies have systematically grouped or assessed the long-term effects of these interventions [10,11,12,13]. In contrast, a number of studies have focused on the short-term impact of resilience-building programs in healthcare employees, including nurses. These studies have been addressed in the recent reviews by Kunzler et al. [1,12], Delgado et al. [10], Henshall et al. [11], and Zhai et al. [13].

Therefore, the aim of this narrative review was to assess the evidence of the long-term impact of resilience-building intervention in nurses by investigating whether research studies evaluating resilience-building interventions included follow-up assessments at least once after the completion of the intervention. The assessment of any effects of resilience-building interventions in the long term is expected to add a new perspective regarding the effectiveness of resilience-building interventions. This is clinically meaningful since it will inform providers of resilience programs for the time frame during which they need to be applied. Potentially, future research can explore what characteristics these interventions need to have in order to be effective longitudinally. Based on these, the secondary objective of this review was to draw implications for critical care nurses.

## 2. Materials and Methods

### 2.1. Study Design

Since the general aim for conducting a narrative review is to summarize and map exiting evidence on a research question in a form that is not explicitly systematic, this study design was applied as the most appropriate for the objectives of this review [14]. Specifically, the literature on the long-term impact of resilience-building programs in nurses has not yet been comprehensively reviewed, while relevant data seems to be heterogeneous and, thus, not suitable for a more precise systematic review [15]. Taking into account the lack of established protocols for conducting a narrative review, the assessment checklist of Baethge et al. [14] was applied herein. According to this protocol, the following research question was set: “What is the long-term impact of resilience building interventions in nurses?”.

A literature search in terms of a systematic and organized manner was conducted from September to 15 November 2024 to identify studies assessing the effectiveness of interventions designed to foster resilience in nurse, with a focus on those that included a long-term follow up period.

### 2.2. Data Sources and Search Strategy

The search was based on the following key-terms alone and in combination: “resilience or psychological resilience”, AND/OR “psychological adaptation” AND “nurses” AND “randomized controlled trial” AND “follow-up”. These terms were applied across multiple databases, encompassing EBSCOhost, MEDLINE, ProQuest, Google Scholar, PubMed, and Scopus. The search was conducted independently by the two authors, and the titles and abstracts of the articles were screened for inclusion and exclusion criteria. There were no disagreements, while cases of uncertainty were resolved with feedback from an expert in medical methodology.

### 2.3. Inclusion and Exclusion Criteria

Studies were included in the review if they met the following criteria:

- Published peer-reviewed Randomized Controlled Trials (RCTs) testing interventions specifically designed to enhance resilience in nurses (RCTs filter);
- Published in English (language filter);
- Measured resilience as an outcome using a standardized, structured, and validated instrument, specific to resilience;
- Included at least one follow-up assessment of resilience beyond the immediate post-intervention period, either as a primary or secondary outcome;
- Full-text access (filter)

There were no publication date-related limitations (no date filter).
The following studies were excluded:

- Studies that engaged nursing students and/or other healthcare professionals;
- Studies that qualitatively assessed the impact of resilience-building interventions;
- Studies that assessed resilience only once immediately after the completion of the intervention;
- Secondary studies and grey literature were not included.

### 2.4. Quality Assessment and Data Extraction

The quality of the included studies was assessed using the guidelines recommended by the Cochrane Collaboration [16], and a summary of the quality assessment results is provided in Table 1. The assessment was conducted independently by the two authors, and disagreements were resolved by consensus. We did not exclude any studies due to insufficient quality.

Aiming to produce a high-level summary of the findings of the included studies, data were extracted based on the variables related to the present research question. These data are presented in Table 2. Subsequently, data extraction was followed by data synthesis in a descriptive and interpretative mode.

### 2.5. Selection of Eligible Studies

A total of 2500 studies were identified from the databases. After removal of dublicates, the titles and abstracts of 1200 studies were screened. Of these, 38 studies assessing the effectiveness of resilience-building interventions in nurses were full-text screened. Next, fifteen studies were excluded because they did not employ a validated resilience measurement tool to assess the impact of the intervention on resilience. Among the remaining 23 studies, only 8 included at least one follow-up assessment of resilience beyond the immediate post-intervention period. Yet, two of these studies were excluded due to insufficient reporting of resilience measurement results at the follow-up intervals. No relevant studies were identified via hand screening, while we did not search any journals by hand.

**Figure 1:**
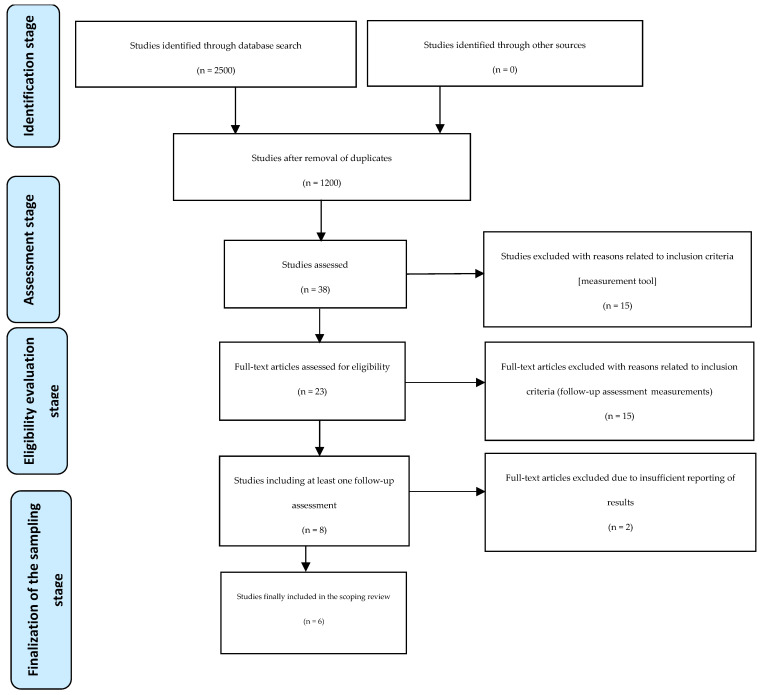
Map of sampling stations located in the Changjiang Ao area of Pingtan Island on 26 April 2022. Abbreviations for the sampling sites are as follows: ABA: Algal Bloom Area, TA: Transition Area, and NBA: Non-Algal Bloom Area.

The selection process of the included studies described by a PRISMA flow chart [15] is presented in Figure 1.

## 3. Results

Six RCTs were included in the review. These studies tested the effect of a resilience-building program exclusively on nurses and reported at least one follow-up assessment of resilience beyond the immediate post-intervention period. The presentation of these studies is summarized in Table 2.

The study by Chesak et al. [17] evaluated the 90 min “Stress Management and Resiliency Training (SMART)” intervention, which was delivered within a nurse orientation program. *A one-hour-long follow-up session* and biweekly topic-related *email handouts* were provided. The control group received a single-session lecture on stress-related topics. Resilience scores improved in the intervention group and declined in the control group at the 3-month follow up. However, the group-by-time interaction, i.e., the resilience score change from baseline to the 3-month follow-up between the two groups, was not statistically significant (*p* = 0.302).

Smith et al. [18] tested the ARISE intervention, designed to support critical care and trauma nurses at a tertiary academic hospital in Toronto, Canada. The intervention consisted of *a full-day workshop* (mindfulness, stress relief through sensory activities, yoga, stretches, and reflective writing), instructor-guided *mindfulness sessions*, and *a half-day follow-up workshop* aiming to reinforce these self-care techniques. Peer support was facilitated via a Facebook group for 3 months. Resilience was measured using the Connor–Davidson Resilience scale (CD-RISC) at baseline, 1 month, and 3 months post intervention. No significant differences in the trajectory of resilience change were observed between the intervention and control group at 1 month (*p* = 0.84) or 3 months (*p* = 0.26).

Lin et al. [19] investigated an *8-week modified mindfulness-based stress reduction (MBSR) program* targeting nurses in two general hospitals in mainland China. Resilience was measured at baseline, immediately post intervention, and at a 3-month follow-up. Although there were no changes in resilience immediately after the intervention, the difference between groups in resilience changes at the 3-month follow-up was statistically significant. Specifically, a significant group-by-time interaction effect was found in this study with nurses in the intervention group demonstrating significantly higher resilience scores at 3 months of follow-up compared to baseline in relation to the control group (*p* < 0.05).

In the study by Grabbe et al. [20] participants in the intervention group attended a *3 h Community Resilience Model (CRM) session*, a sensory-focused mindfulness program. The control group attended a 3 h class on nutrition and healthy eating. Resilience was measured at baseline, one week post intervention, and at three months and one year. Although there was no significant group-by-time interaction, a significant time effect was observed in the CRM intervention group at 3 months, indicating moderate improvement in resilience (effect size, d = 0.39). No improvements were reported at one week or one year.

Janzarik et al. [21] examined an *8-week psychotherapy program* for nurses working in various departments of a large German hospital. Assessments were conducted at baseline (t0), immediately (one week) post intervention (t1), and three (t2), six (t3), and nine months (t4) post intervention. The intervention group experienced significant improvement in resilience, with fewer mental health problems reported, compared to the control group. However, while group differences in resilience were statistically significant for follow-up assessments using the CD-RISC, no statistically significant differences were found when resilience was measured with the BRS.

The “Promoting Resilience in Nurses©” program was implemented by Foster et al. [22], in a large tertiary, mental health service in Australia. The six-module program was delivered in nurses by accredited facilitators. Resilience, measured using the six-item Brief Resilience Scale (BRS), was found to be statistically and significantly improved post intervention, with greater group-by-time effect at the 3-month follow-up compared to the immediate post-intervention period.

## 4. Discussion

The present study aimed to evaluate the long-term effects of resilience-building interventions in nurses, as measured via RCTs. This is particularly relevant, given the review by Kunzler et al. [12], which emphasized the importance of longer follow-up periods to better understand the efficacy of psychological interventions aiming to foster resilience. We identified only six studies that included long-term follow-up assessment of resilience. Of the six studies identified, five applied the CD-RISC tool as a resilience measurement tool, all of which demonstrated improvements in resilience scores at the 3-month follow-up [17,18,19,20,21]. Thus, one study, which solely used the BRS, reported statistically significant improvements of resilience at the 3-month follow-up [22]. However, in the study by Janzarik et al. [21] significant improvements in resilience at the 3-month follow-up were observed using the CD-RISC tool, but not the BRS.

This discrepancy is notable, as the CD-RISC tool focuses on assessing the availability of resilience factors, whereas the BRS evaluates resilience as an outcome [23]. These findings suggest that resilience development involves complex interactions between acquired skills and the capacity to apply them, which may evolve over time. Furthermore, the lack of sustained effect at longer follow-ups, such as one year, highlights the potential need for periodic reinforcement sessions to sustain resilience gains.

Our findings are supportive of the model proposed by Bonnano et al. [24], which examines resilience as a response over the course of time. Four elements are important in the appreciation of the temporal characterization of resilience: (a) baseline adjustment; (b) aversive circumstances; (c) post adversity resilient outcomes; and (d) predictors of resilient outcomes measured prior to, during, and after the aversive circumstances. For this reason, measuring resilience after an intervention requires longitudinal designs involving repeated and frequent monitoring of mental health, stressor exposure, and potential adaptations. The six studies included in this review, although limited in number, attest to the integration by investigators of the temporal approach in the study of variations in resilience after interventions. Assessing the outcomes in several timepoints after the completion of the intervention provides interesting information for the effectiveness of the intervention and the potential need for improvements according to different temporal adaptations of participants. Cross-sectional studies might overlook distinct trajectories of different subgroups on participants [9]. Also, despite previous reports of resilience interventions in nursing, only a few attempts to group and assess the long-term effects of resilience-building interventions are available [10,11,12,13].

Nevertheless, the present findings must be interpreted cautiously due to the limited number of included studies and the methodological heterogeneity among them. For instance, two of the studies employed longer interventions lasting 8 to 12 weeks [19,21], while others used shorter interventions, such as a single 90 min lecture [17], a three-hour-long session [20], or a full-day workshop [18]. It is worth mentioning that studies with longer interventions (i.e., beyond a single session) appear to have slightly more favorable results. Assuming this finding is confirmed in future studies, it will attest to the need for ongoing support for nurses. However, although Lin et al. [19] implemented longer interventions and employed a waitlist control group, this has been noted to potentially overestimate treatment effects, warranting careful evaluation of the conclusions drawn [25].

The present findings align with previous reviews, which have shown that while resilience-building interventions may enhance resilience immediately post-intervention, they often fail to produce lasting improvements in well-being [1,12]. Similarly, Zhai et al. [13], in their meta-analysis, concluded that none of the reviewed studies provided sufficiently extended follow-up assessment to capture meaningful long-term effects. Future studies should prioritize long-term measurement intervals to more accurately assess the sustained impact of resilience interventions.

Additionally, most programs reviewed focused solely on individual-level resilience, overlooking the broader environmental and organizational context. The review by Kunzler et al. [12] faced similar criticism for drawing conclusions from interventions that failed to address resilience as a dynamic response influenced by environmental factors [26]. Organizational involvement and support are essential to enhance lasting resilience in healthcare professionals.

Despite the growing body of literature on resilience interventions in nursing [10,11,12,13], only a few studies specifically targeted critical care nurses. Exceptions include three pre-post studies [18,23,27], of which only one included a longer-term follow-up assessment [18]. In that study, no significant changes in resilience scores (CD-RISC) were reported between the intervention and control groups at 1 or 3 months post invention. The other two studies assessed resilience only immediately after the intervention [23,27], finding improvements in resilience scores that may not persist without follow-up data. The interventions in these studies varied widely, ranging from a full-day workshop [18] to multi-session programs involving educational components workshop, counseling, mindfulness-based stress reduction, and exercise regimens delivered over 12 weeks [23], or five 90–120 min sessions covering resilience principals and development strategies [27].

Lastly, the present review underscored a critical gap in the literature: the lack of comprehensive assessments of individual risk for developing mental disorders following severe stress exposure. Vanhove et al. [28] observed that resilience interventions provided universally to employees, regardless of their risk profile, tend to have effects that peak immediately after training and diminish over time. In contrast, interventions targeted at high-risk employees demonstrate increasing effects over time, with greater benefits observed at the later follow-ups compared to immediately post intervention [28]. Future research on resilience-building in nurses should integrate individual risk assessment for mental health vulnerabilities and prioritize providing tailored support for those at higher risk.

### Limitations

Despite the valuable insights provided by this scoping review, its findings should be interpreted with caution due to the limited number of included studies and their methodological heterogeneity. These factors also limited the ability to perform a more sophisticated synthesis of the data. Indeed, the substantial variability among the reviewed studies poses significant challenges for direct comparisons of the programs, particularly regarding their content, duration, teaching methods, and the instructors’ expertise and preparation. The use of multimodal interventions further complicates the ability to determine which specific components contributed to each program’s efficacy. Additionally, methodological limitations of the primary studies affect the certainty and generalizability of the evidence in this review. One more notable limitation of the reviewed studies was the scarcity of data extending beyond three months post intervention, which restricts conclusions about the stability of observed effects over time and the potential need for booster sessions. Furthermore, the studies were constrained by small sample sizes and predominantly female participants, which limits the applicability of findings to a more diverse nursing population.

The exclusivity of RCTs and the restriction to publications in English represent additional limitations, as they may exclude relevant evidence available in other formats or languages. Also, the absence of data on potential work-related factors, such as salary and shift work may be a limitation too. These unaccounted variables overlook the critical role that the work environment may constitute in influencing nurses’ adaptive or maladaptive responses to stress and, subsequently, to resilience outcome. To address this, future research should evaluate environmental variables when assessing the efficacy of educational programs [29]. It is possible that resilience training alone may not be sufficient to achieve sustainable outcomes in a work environment that fails to meet the minimum standards of clinical practice [30]. As Kyranou [31] suggested, the broader clinical setting must be conducive to foster resilience. An additional limitation regards the fact that two studies were excluded from the present review due to insufficient reporting of the tools/methods applied in the post-intervention measurements, with no attempt to obtain these data from the researchers. Yet, since the general aim for conducting a narrative review is to summarize and map exiting evidence on a research question in a form that is not explicitly systematic, this limitation is not expected to crucially disempower the present findings. These highlight the need for future studies assessing the long-term impact of resilience-building interventions, also underscoring crucial points in designing such studies.

## 5. Conclusions

This narrative review highlights the very limited number of studies examining the long-time effects of resilience training in nurses, particularly in critical care settings. Some preliminary evidence points to the absence of sustained effect at longer follow-ups. As a result, professionals designing resilience-building interventions need to appreciate potential attrition effects not only in the number of people applying the intervention but also in the effectiveness of the intervention itself over time. Future studies could explore whether the provision of “booster” sessions, after the completion of the program, can improve resilience outcomes in the long run.

Interventions aimed at alleviating the stress experienced by hospital nurses and enhancing their resilience are likely to be pivotal in efforts to retain and support the nursing workforce. This is especially true in critical care settings, where the ever-changing and high-pressure nature of the work environment significantly amplifies occupational stress. A promising approach is the development of interventions that address both individual and organizational factors over time. Longitudinal strategies that integrate personal resilience training with systemic changes in the workplace environment may be more effective in promoting adaptive responses to severe or chronic work-related stress. Such comprehensive interventions could play a crucial role in building and sustaining resilience among nurses in the challenging context of healthcare settings.

## Figures and Tables

**Table 1:** Risk of bias assessment of the included studies (Cochrane tool).

Included Studies	Random Sequence Generation (Selection Bias)	Allocation Conceleament (Selection Bias)	Blinding of Participants and Personnel (Performance Bias)	Blinding of Outcome Assessment (Detection Bias)	Incomplete Outcome Data (Attrition Bias)	Selective Reporting (Reporting Bias)
[17]	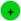	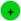	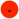	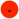	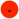	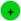
[18]	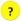	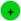	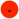	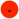	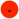	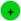
[19]	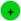	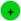	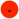	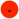	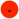	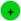
[20]	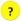	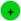	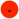	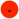	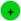	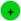
[21]	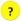	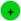	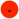	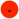	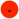	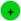
[22]	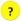	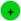	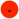	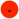	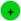	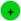
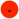 = High risk 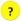 = Unclear risk 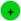 = Low risk						

**Table 2:** Randomized Controlled Studies included in the review in chronological order.

Authors, Year, Country	Aim/Setting and Participants	Intervention Description	Conceptualization of Resilience/Tool	Assessment/Findings				
				Pre-Intervention	Post-Intervention			
				Baseline Assessment	Right after the completion of the program	Short-term follow-up (3 months) **(mean** ± **SD)**	Long-term follow-up (6 months) **(mean** ± **SD)**	Long-term follow-up (9–12 months) **(mean** ± **SD)**
[17], 2015, USA	**Aim:** Examination of the outcomes of a brief Stress Management and Resiliency Training (SMART) program within a nurse orientation program. **Setting/Population**: Nurse orientation program at Mayo Clinic **Sample size**: 55 - **Intervention**: n = 27 - **Control**: n = 28 **Age** (mean) = 28.16 years (SD: 8.29) **Sex**: 38 women, 2 men	*Intervention* **Delivery:** face-to-face lecture. **Description**:Presentation of a model of stress and resilience, integrating neuroscience and biology. **Duration of treatment period and timing**: Single 90 min session; 1 h follow-up session after 4 weeks; bi-weekly handouts via email. *Control* **Delivery:** Lecture **Description**: Lecture related to stress, including reality shock and work–life connectedness. **Duration of treatment**: Single session.	SMART is designed to help participants understand the neuroscience and biology of stress. Resilience is seen as a set of factors whose modification can be achieved using the 5 core principles of gratitude, compassion, acceptance, forgiveness, and higher meaning. Connor–Davidson Resilience Scale	*Intervention group* 79.68 ± 9.59 *Control group* 74.76 ± 10.19		*Intervention group* 79.74 ± 11.82 *Control group* 72.52 ± 8.83		
				A two-sample *t* test comparing the change from baseline between groups found an estimated treatment effect (95% CI): 0.29 (2.15, 6.73) (*p* = 0.302).				
[18], Canada	**Aim**: To test the effects of a wellness intervention (ARISE) on resilience, professional quality of life, and other outcomes, in critical care and trauma nurses. **Setting/Population:** A tertiary academic hospital in Toronto, Canada. **Sample size:** 29 nurses - **Intervention group**: n = 16 - **Control group**: n = 13 **Age** (mean): 33 years **Sex:** 90% female **Work experience in nursing** = 7.6 years	The intervention consisted of: (1). One full-day interactive workshop with resilience-focused activities and (2). Self-care techniques including mindfulness, stress relief using senses, yoga and stretches, and reflective writing, and a half-day interactive workshop which reinforced self-care techniques and introduced hospital resources for wellness and employee and family assistance. A closed Facebook group was used to bolster workshop content workshops and provide peer support for 3 months. Five 90 min instructor-guided mindfulness sessions were offered and archived for repeated use.	The CD-RISC was used to measure resilience at baseline, and 1 and 3 months after the intervention.		There was no difference in the change in CD-RISC scores from baseline between the intervention and control groups at 1 month (*p* = 0.84).	There was no difference in the change in CD-RISC scores from baseline between the intervention and control groups at 3 months (*p* = 0.26).	NA	NA
[19], China	**Aim:** To evaluate the effects of a modified mindfulness-based stress reduction (MBSR) program on the levels of stress, affect, and resilience among nurses, in general, hospitals in mainland China. **Setting/Population**: nurses from two tertiary-level general hospital **Sample size**: 110 nurses - **Intervention group**: n = 55 - **Wait-list Control**: n = 55 **Age (mean)**: 31.50 years (SD: 6.90) **Sex**: 84 women, 6 men	*Intervention* **Delivery:** Face-to-face group sessions **Description**: Guided practice, education, dialogs around participants’ observations of their feelings, thoughts, and body sensations. **Duration of treatment period and timing**: Eight 2-h weekly group sessions. 20 min of formal mindfulness practice at home daily for 6 days a week for 8 weeks. Network Chatgroup through “WeChat”on mobile phones (sending PowerPoint slides, audio recordings of mindfulnessexercises, questions and answers). *Wait-list Control* They were offered the intervention at a later date.	Modified mindfulness-based stress reduction (MBSR) program Resilience is seen as a set of factors whose modification can be achieved with training Connor–Davidson Resilience Scale	*Intervention group* 54.43 ± 11.46 *Control group* 55.17 ± 11.85	*Intervention group* 57.98 ± 11.58 *Control group* 55.11 ± 12.80	*Intervention group* 59.70 ± 11.87 *Control group* 53.85 ± 16.21	NA	NA
				Significant differences in resilience were found between the two groups at the 3-month follow-up (*t* = 1.95, F = 4.587, *p* < 0.05).				
[20] USA	**Aim:** Investigation of the effectiveness of a short resiliency intervention focusing on sensory awareness on stress and resilience. **Setting/Population**: Registered nurses at two tertiary care hospitals (including emergency department, operating room, intensive care units, specialty units, out-patient clinics, medical-surgical units) **Sample size**: 196 - **Intervention**: n = 99 - **Control**: n = 97 **Age** (mean: 45.3 years (SD: 13.2) **Sex**: 95% women Of the 196 nurses who completed baseline surveys and were randomized into treatment or control groups, only 77 (39.3%) came to a class and completed one or more of the follow-up surveys.	*Intervention* **Delivery:** Face-to-face lecture, active engagement, discussion, demonstration, and participation, and various active experiences, such as skills stations. Access to the free CRM “ichill” app after the class. **Description**: Psychoeducation/sensory awareness skills training class. **Duration of treatment period and timing**: 3 h lecture *Control* **Delivery:** face-to-face lecture, active engagement, discussion, demonstration, and participation, and various active experiences, such as mindful eating. Access to the free CRM “My Plate” app after the class. **Description**: Lecture that covered topics related to nutrition/healthy eating. **Duration of treatment**: 3-h lecture.	Trying to achieve the “Resilient Zone”, an internal state of balance. Resilience is seen as a set of factors whose modification can be achieved with training. Connor–Davidson Resilience Scale	*Intervention group* 29.65 ± 5.43 *Control group* 28.82 ± 11.85	*Intervention group* 30.42 ± 3.35 *Control group* 30.22 ± 5.32	*Intervention group* 30.94 ± 4.07 *Control group* 31.00 ± 5.16	NA	*Intervention group* 31.72 ± 4.02 *Control group* 30.54 ± 4.99
				Resilience significantly improved over time (*p* = 0.004). Post hoc tests performed for time within each group revealed significant effects for resilience (F(3, 193.8) = 2.689, *p* = 0.048), with no significant time efects for the control group (*p* > 0.10).				
[21] Germany	**Aim:** Assessment of the improvement of mental health problems assessed by the General Health Questionnaire-28. Secondary outcome measures included self-reported indicators of resilience. Subjects were randomly assigned to either an intervention or a waiting list control group. **Setting/Population**: Nurses working in various medical departments (e.g., neurology, surgery, dermatology, etc.) and 30% critical care nurses in both groups. **Sample Size**: 72 nurses - **Intervention group**: n = 38 - **Control group**: n = 34 **Age:** Intervention group mean = 47.4 (SD:10.8) years Control group mean = 46.5 (SD:10.4) years **Sex** Intervention group 35 women, 3 men Control group 31 women 3 men	**Intervention:** an 8 weekly sessions of psychotherapy, two hours each held in group. G.J., a psychologist in post-graduate psychotherapist, conducted all training sessions. Cognitive behavioral therapy and psychodynamic and imagination exercises were included. No blinding was involved in the study.	Self-reported indicators of resilience assessed with: - The Brief Resilience Scale (BRS) - the Connor–Davidson resilience scale(CD-RISC). While the BRS conceptualizes resilience as the ability to recover from stress, the CD-RISC operationalises resilience as a composite of resilience factors.	(t0) was conducted one week before the first training session *Intervention group* (BRS) 3.08 ± 0.34 (CD-RISC) 70.40 ± 11.84 *Control group* (BRS) 2.85 ± 0.50 (CD-RISC) 69.88 ± 11.85 *70–73 &*	(t1) within one week after the last training session. *Intervention group* (BRS) 3.12 ± 0.32 (CD-RISC) 73.436 ± 12.38 *Control group* (BRS) 2.85 ± 0.50 (CD-RISC) 69.33 ± 12.35	(t2) three months *Intervention group* (BRS) 3.73 ± 0.65 (CD-RISC) 72.03 ± 11.89 *Control group* (BRS) 3.45 ± 0.83 (CD-RISC) 69.81 ± 13.57	(t3) six months *Intervention group* (BRS) 3.80 ± 0.58 (CD-RISC) 73.80 ± 12.75 *Control group* (BRS) 3.57 ± 0.78 (CD-RISC) 69.43 ± 13.16	(t4) nine months *Intervention group* (BRS) 3.79 ± 0.59 (CD-RISC) 71.12 ± 12.23 *Control group* Due to the beginning coronavirus pandemic, the control group could not take part in the last measurement and received the intervention after t3
				The mixed model analysis revealed statistically significant group effects for the follow-up period (t1–t4) for resilience measured with the CD-RISC (β = 3.46, *t* = 2.14, *p* = 0.04). No statistically significant group differences for the follow-up period were found for resilience measured by BRS (β = 0.07, *t* = 0.66, *p* = 0.51).				
[22], Australia	**Aim:** To determine the effects of a resilience-building program on mental health nurses’ coping self-efficacy (primary outcome), and psychological distress, well-being, resilience, posttraumatic growth, emotional intelligence behaviors, workplace belonging, and turnover intention (secondary outcomes). **Setting/Population**: Registered and enrolled nurses working in clinical roles in a large tertiary metropolitan mental health service. **Sample size**: 144 nurses - **Intervention**: n = 73 - **Control**: n = 71 **Age** groups Intervention group 20 to 29, 25 (37%) 30 to 39, 22 (32%) 40 to 49, 13 (19%) 50+, 8 (12%) Control group 20 to 29, 17 (25%) 30 to 39, 26 (39%) 40 to 49, 9 (13%) 50+, 15 (22%) **Sex**: Intervention group 54 women (74%) 19 men (26%) Control group 50 women (70%) 20 men (28%)	**Content -Six modules** identifying strengths and understanding resilience; understanding and managing stress; challenging and changing negative self-talk; drawing strength from adversity; promoting positive relationships and managing conflict; and creating solutions for well-being. **Teaching methods** Two 1-day workshops, delivered three weeks apart, and receive booster activities. Workshops were delivered face-to-face by two trained facilitators (experienced senior mental health nurses) in a peer-group setting. The program is using various teaching modalities including video clips, didactic sessions, small and large group discussions, and individual activities. Participants receive ‘booster’ activities via Short Message Service weekly, 10 min to complete. Delivery of the intervention comprised 23 individuals content units across the six modules. Fidelity checklists were completed by facilitators for each program, with each content unit assessed as: (1) Yes, delivered in full, (2) Yes, delivered in part, or (3) No.	Promoting Resilience in Nurses© program is underpinned by interpersonal theory, cognitive behavior theory and posttraumatic growth theory. Resilience was measured with the 6-item Brief Resilience Scale (BRS)	*Intervention group* (BRS) 3.37 ± 0.66 *Control group* (BRS) 3.54 ± 0.74	Estimates and 95% confidence intervals for resilience, expressed as intervention minus control. 0.24 (0.01 to 0.46) (*p* = 0.04)	Estimates and 95% CI for resilience, expressed as intervention minus control. 0.30 (0.08 to 0.52) (*p* = 0.009)	NA	NA
				Resilience improved and was sustained at 3 months with sighltly smaller T3 estimates than T2.				

## Data Availability

No new data were created.
